# Risk Assessment and Potential Analysis of the Agricultural Use of Sewage Sludge in Central Shanxi Province

**DOI:** 10.3390/ijerph19074236

**Published:** 2022-04-01

**Authors:** Baoling Duan, Qiang Feng

**Affiliations:** College of Resources and Environment, Shanxi University of Finance and Economics, Taiyuan 030006, China; sxcddbl@sxufe.edu.cn

**Keywords:** heavy metals, monomial potential ecological risk coefficient, soil environmental capacity, potential ecological risk index, agricultural use potential of sewage sludge

## Abstract

The agricultural use of sewage sludge has become an economic disposal method used worldwide. However, heavy metals contained in sewage sludge have become the crucial limiting factors for this way of disposal. This study showed that regulatory limit values are not enough to determine whether sewage sludge is suitable for agricultural use; risk assessment and potential analysis should be applied. Correlation analysis and hierarchical cluster analysis (HCA) should also be performed to identify heavy metals’ sources and show their influence on sewage sludge utilization. Samples were collected from 13 wastewater treatment plants (WWTPs) located in central Shanxi Province. Results indicated that the mean contents of heavy metals in sewage sludge were all less than the limit threshold of China. According to the monomial potential ecological risk coefficient ($E_{r}^{i}$), the agricultural use of sewage sludge had low ecological risks for all heavy metals, except for Hg and Cd. Based on the potential ecological risk index (RI), only three stations had moderate risk, other nine stations all had higher potential risk. The mean potentials by all heavy metals were all beyond 10 years, which is the limit of the maximum application time specified by China. Combining all heavy metals, only one station’s potential was less than 10 years. Although the contents of heavy metals were all within the threshold values, large quantities of sewage sludge are not suitable for agricultural use. Coal-related industries, which were the main sources of Hg and Cd, greatly affected the agricultural use of sewage sludge.

## 1. Introduction

After the prohibition of direct sea dumping in most countries, sewage sludge is generally disposed of by land filling, incineration, and land application [1,2,3,4,5]. Due to the abundance of organic matter, nitrogen, phosphorus, potassium, and other nutritional elements that are necessary for plant growth, land application is regarded as an economic way to dispose of sewage sludge worldwide [6,7,8,9]. In the United States, approximately 60% of sewage sludge is used as a soil conditioner [3,10]. In Europe, more than 40% of sewage sludge is used on agricultural land [3,11]. In China, the proportion of the agricultural use of sewage sludge is 48.28% [12]. In Shanxi, approximately 42.66% of sewage sludge is disposed of by land use [13].

Since the properties are nondegradable, heavy metals are persistent toxic pollutants. Once heavy metals enter the environment, they will chronically exist and constantly accumulate [14]. When sewage sludge is utilized on agricultural land, harmful substances, such as heavy metals contained in it, will enter the soil [15]. Because of the connectivity of ecosystems, heavy metals entering soil accompanied by the agricultural use of sewage sludge, will also cause heavy metals to enter other ecosystems, such as the atmosphere, groundwater, surface water, and biosphere [16,17,18,19]. In addition to posing threats to the ecological environment, the heavy metals contained in sewage sludge can also pose risks to human health through groundwater, the human food chain, and other ecosystems [16,17,20]. Therefore, the heavy metals in sewage sludge have become an important limiting factor for the use of sewage sludge [21]. To safely use sewage sludge and prevent the adverse effects induced by heavy metals, studies generally have focused on the distribution of heavy metals’ contents and ecological and human health risk assessments [22,23,24,25,26]. However, to be effective and safe, the disposal method for sewage sludge should be selected not only based on the contents of heavy metals and risk assessment but also by adding potential analysis. The purpose of this paper was to briefly introduce potential analysis, which can clearly show the service life of sewage sludge, and to assess the influence of heavy metals’ sources on sewage sludge utilization. This can provide a scientifically based reference for reducing the risk of the agricultural use of sewage sludge, while at the same time improving the resource utilization level.

The total quantity and the proportion of the agricultural use of sewage sludge in central Shanxi Province are higher than those in other regions of this province. In this central region, a total amount of 127,950.2 tons of sewage sludge is disposed of by land application, and the ratio is 72.90%, which is much higher than that of other disposal methods [13]. The aims of this study were to (1) determine the contents of the heavy metals in sewage sludge sampled from different wastewater treatment plants (WWTPs) located in central Shanxi Province, (2) assess heavy metals’ ecological risk when sewage sludge is applied on agricultural land, (3) calculate the soil environmental capacity of different heavy metals, and (4) measure the potential of the agricultural use of sewage sludge.

## 2. Materials and Methods

### 2.1. Studying Area

Shanxi Province, located in the Loess Plateau, has poor soil. The contents of organic matter, total nitrogen, phosphorus, and potassium of cultivated land in this province are lower than those in other regions of China. As the biggest coal base in China, the pillar industries are coal and coal-related industries such as coke, metallurgy, electricity, and chemical production; these industries affecting heavy metals’ contents in sewage sludge is typical in China.

### 2.2. Sampling and Chemical Analysis

Sewage sludge samples were collected from different WWTPs located in central Shanxi Province. To enhance the representation of each sample, subsamples were collected from four different sites in each WWTP and then combined, forming a single sample.

The samples were dried in a clean environment at room temperature. Next, they were sieved through a 0.14-mm mesh screen and stored in brown glass bottles [27]. Then, samples were weighed and digested with HNO_3_ using a microwave digestion system based on the USEPA Method 3051B [28]. Cu, Zn, Pb, and Cr were analyzed using atomic absorption spectrophotometer. Cd was analyzed using a graphite furnace atomic absorption spectrophotometer. As was analyzed using atomic fluorescence spectrometer. Certified sewage sludge samples and blank samples were tested. To control the quality, the Chinese national standards GB/T 15555.2-1995, GB/T 15555.2-1995, GB/T 15555.2-1995, GB/T 15555.6-1996, GB/T 17141-1997, and GB/T 22105.2-2008 were used. 

### 2.3. Ecological Risk Assessment

In 1980, the potential ecological risk index (RI) proposed by Hakanson was widely applied to assess heavy metal pollution [29]. This method evaluates the ecological risk of heavy metals based on their accumulation, toxicity, and environmental behavior. The equations are as follows.

(1) $$C_{f}^{i}=C_{s}^{i}/C_{n}^{i}$$

(2) $$E_{r}^{i}=T_{r}^{i}\times C_{f}^{i}$$

(3) $$\mathrm{RI}=\sum E_{r}^{i}$$

where $C_{f}^{i}$ is the single heavy metal pollution factor of the ith heavy metal; $C_{s}^{i}$ is the content of the ith heavy metal in the samples, mg/kg; $C_{n}^{i}$ is the background values of the ith heavy metal, according to the soil heavy metal background values of Shanxi Province; the corresponding standard values for Cu, Zn, As, Hg, Pb, Cd, and Cr are 22.9 mg/kg, 63.5 mg/kg, 9.1 mg/kg, 0.023 mg/kg, 14.7 mg/kg, 0.102 mg/kg, and 55.3 mg/kg, respectively; [30]; $E_{r}^{i}$ is the monomial potential ecological risk coefficient of the ith heavy metal; $T_{r}^{i}$ is the heavy metal toxic response factor, according to Hakanson; the values for each heavy metal are Zn (1) < Cr (2) < Cu (5) = Pb (5) < As (10) < Cd (30) < Hg (40) [31]; and RI is the potential ecological risk index.

Contamination from heavy metals is classified into five levels according to the $E_{r}^{i}$ as follows: $E_{r}^{i}$ < 40, low risk; 40 ≤ $E_{r}^{i}$ < 80, moderate risk; 80 ≤ $E_{r}^{i}$ < 160, high risk; 160 ≤ $E_{r}^{i}$ < 320, very high risk; and $E_{r}^{i}$ ≥ 320, extremely high risk. The classification of heavy metal pollution according to RI is as follows: RI < 150, low risk; 150 ≤ RI < 300, moderate risk; 300 ≤ RI < 600, high risk; and RI ≥ 600, very high risk [32,33,34].

### 2.4. Soil Environment Capacity of Heavy Metal

The soil environment capacity of heavy metal is the maximum load of heavy metal undertaken by a soil and under which the yield and biological quality of agricultural products can be guaranteed [35]. The soil environment capacity of heavy metal is also an indicator of the ability of a soil to accommodate a particular heavy metal yet not be polluted by it [36]. If the additive amount exceeds this amount, the soil environment will be exposed to heavy metal pollution, which can cause environmental pollution and further threaten human health [35]. The calculation of it is as follows [37].

(4) $$Q_{i}=2.25\times 10^{6}\times \left(S_{i}-C_{i}\right)\times 10^{-6}$$

where $Q_{i}$ is the soil environment capacity of the ith heavy metal, kg/km^2^; $S_{i}$ is the standard value of the ith heavy metal, mg/kg; the risk screening value for soil contamination of agricultural land in Soil Environmental Quality–Risk Control Standard for Soil Contamination of Agricultural Land (GB 15618-2018) is used as the standard value; $C_{i}$ is the background value of the ith heavy metal for the national soil (layer A) of Shanxi Province, mg/kg; 2.25 × 10^6^ is the average quality of per hectare, kg; and 10^−6^ is the conversion coefficient. 

### 2.5. Agricultural Use Potential of Sewage Sludge

The potential of the agricultural use of sewage sludge is represented by the years of safe land application, which is evaluated based on the threshold value of the soil environment capacity of heavy metal. The equation is as follows [37].

(5) $$N_{i}=\frac{Q_{i}}{7500\times C_{s}^{i}}\times 10^{6}$$

(6) $$N={\mathrm{MIN}}_{N_{i}}$$

where $N_{i}$ is the agricultural use potential of sewage sludge for the ith heavy metal when the ith heavy metal contained in sewage sludge reaches its soil environment capacity, a; 7500 is the maximum application amount of sewage sludge for per year and per hectare based on the Disposal of Sludge from municipal Wastewater Treatment Plant–Control Standards for Agricultural Use (CJ/T 309–2009), kg/(km^2^ × a); 10^6^ is the conversion coefficient; and N is the agricultural use potential of sewage sludge, a.

### 2.6. Data Analysis

Statistical analyses were performed by Microsoft Office Excel 2019 (Microsoft Corporation, Redmond, WA, USA) and SPSS 20 (IBM Corp., Armonk, NY, USA). Descriptive statistics (maximum, minimum, average, and standard deviation) were calculated. The relationships between the content of each heavy metal in sewage sludge were identified by Pearson correlation analysis; this is an important demonstrator of heavy metals’ sources. The correlation coefficient near 1 manifests a strong correlation between heavy metals. Hierarchical cluster analysis (HCA) was also used to determine the correlation between individual heavy metals and to confirm the sources.

## 3. Results

### 3.1. The Heavy Metal Content in Sewage Sludge

The contents of heavy metals in sewage sludge are presented in Table 1. The results indicated a general trend in the contents of heavy metals in sewage sludge: Cu > Zn > Cr > Pb > As > Hg > Cd. On the mean, concentrations of Cu, Zn, Cr, Pb, As, Hg, and Cd were 175.99 mg/kg, 146.03 mg/kg, 126.68 mg/kg, 45.09 mg/kg, 15.1 mg/kg, 2.85 mg/kg, and 2.71 mg/kg, respectively. Cu was the most abundant heavy metal in sewage sludge; Cd was the least abundant. 

The heavy metals in the sewage sludge had significant variations, and the general trend was Zn > Cu > Cr > Pb > Cd > As > Hg. Contents of Zn, Cu, Cr, Pb, Cd, As, and Hg varied from 52.27 mg/kg to 855.00 mg/kg, 88.62 mg/kg to 261.00 mg/kg, 33.29 mg/kg to 665.31 mg/kg, 26.80 mg/kg to 57.39 mg/kg, 0.331 mg/kg to 7.23 mg/kg, 7.87 mg/kg to 23.40 mg/kg, and 0.88 mg/kg to 5.11 mg/kg, respectively. Because the sewage sludge samples were collected from different regions, the contents of the heavy metals in sewage sludge varied greatly [38]. Specifically, the heavy metals in sewage sludge are impacted by many factors, such as industry, traffic, households, water supply systems, stormwater, drainage, and leakage water, but the main source apportionment is an anthropogenic component, which can greatly affect the contents of heavy metals in sewage sludge [28,38,39].

**Table 1:** Contents of the heavy metals in the sewage sludge from different WWTPs located in central Shanxi Province (mg·kg^−1^).

Station	Cu	Zn	As	Hg	Pb	Cd	Cr
1	261.00	63.44	14.39	3.74	57.39	0.45	186.24
2	244.87	107.16	17.61	2.88	41.13	1.06	91.55
3	149.94	86.98	13.85	3.72	55.55	0.80	131.80
4	160.19	121.10	15.09	1.93	56.53	0.74	54.48
5	254.39	89.51	22.51	1.72	43.57	0.33	93.66
6	129.84	66.84	14.84	5.11	37.42	0.59	71.57
7	180.55	81.46	15.94	4.12	51.09	0.96	665.31
8	146.79	99.29	16.92	3.08	44.64	10.57	65.23
9	88.62	86.17	12.73	4.38	34.24	0.36	41.27
10	256.65	105.75	8.99	2.48	42.59	0.88	48.99
11	170.03	83.43	12.13	1.50	51.77	0.76	41.71
12	138.35	52.27	7.87	1.52	43.43	0.55	33.29
13	106.60	855.00	23.40	0.88	26.80	17.23	121.70
Min	88.62	52.27	7.87	0.88	26.80	0.33	33.29
Max	261.00	855.00	23.40	5.11	57.39	17.23	665.31
Mean	175.99	146.03	15.10	2.85	45.09	2.71	126.68
SD	109.18	559.72	6.37	2.02	21.63	11.87	45.63

**Figure 1:**
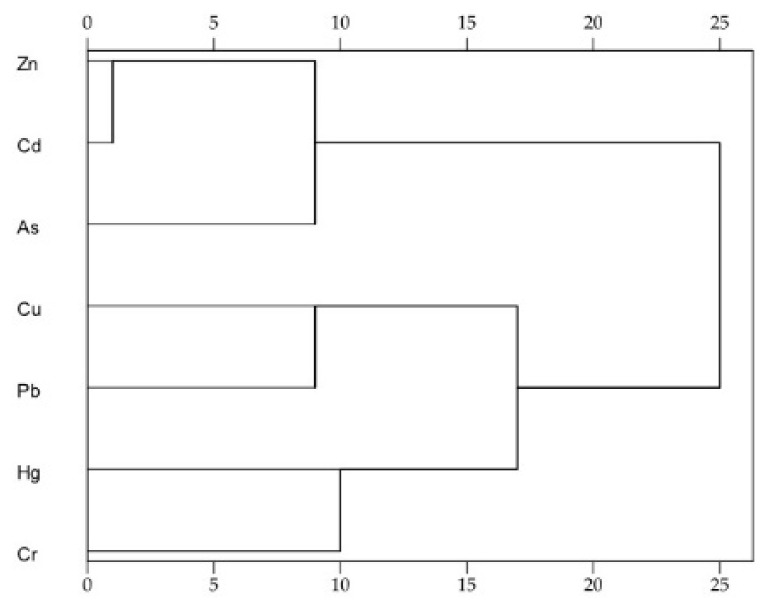
HCA dendrograms for sewage sludge samples.

Pearson correlation analysis was performed (see Table 2); a higher correlation coefficient between different heavy metals indicated they might have the same sources. In this study, highly significant, positive correlations were observed between Zn and As, Zn and Pb, Zn and Cd, and As and Cd. The significant correlations between Zn-As-Pb-Cd and As-Cd revealed that they may come from the same source. This relationship between heavy metals is the same as in other studies [18,40,41,42]. HCA was also applied to identify the sources of heavy metals contained in sewage sludge (as in Figure 1); the dendrogram based on the contents of heavy metals was divided into three groups. Zn, Cd, and As were the first group; Cu and Pb were the second group; and Hg and Cr were the last group. This result was basically consistent with correlation analysis. Combining with the local industries, the pollutions of Zn, Cd, and As were identified as malleable cast iron production, Cu and Pb were induced mainly by steel smelting and traffic, and Hg and Cr were caused by coal-related industries. At station 1, the contents of Cu and Pb were the highest, which might have been induced by the largest steel smelting industry of Shanxi Province being located in this area [43,44]. At Station 13, the largest contents of Zn, As, and Cd might have been because this station is one of the malleable cast iron production bases of China [45]. At Station 6, due to the production of gold jewelry and the first gold jewelry culture industrial park in Shanxi Province, the content of Hg was the highest [46,47]. At Station 7, in which the chemical engineering industry and textile industry are located, the contamination of Cr was the most serious [48,49]. Because Stations 1, 3, 4, 7, and 11 are traffic transportation junctions, samples collected from these stations had high concentrations of Pb, which was caused by the use of leaded gasoline [5,50,51,52]. The pollution by Hg was high at Stations 1, 2, 3, 7, 8, and 9, which might have been caused by the coal-related industries such as the steel smelting industry, coal chemistry industry, and coking industry [44,48].

To reduce soil and groundwater pollution, the limit values of heavy metals in sewage sludge for agricultural use are formulated by different countries. Referring to international standards and combining with the actual local condition, China has also developed corresponding standards. As shown in Table 3, the limit values of China are basically consistent with that of the US and most EU countries, except in Canada where the limit of As is less than the value established by other countries. However, in some of EU countries, such Austria and the Netherlands, the management of land application is improper due to the restrictions or prohibitions.

Due to the background pH value in the study area being 8.4, which can be compared with the limit values stipulated by the Chinese Discharge Standard of Pollutants for Municipal Wastewater Treatment Plant (GB 18918–2002) for pH ≥ 6.5, the maximum contents of the heavy metals in the samples were all below limit values, except for the As content, which was higher than the limit value established by Canada. This indicated that in this study area, with the exception of As, the heavy metals’ contents in sewage sludge were not high.

Comparing the mean values of the study area with those of China, the contents of As, Hg, Cd, and Cr were higher than those of China, which was mainly due to the steel smelting industry, malleable iron industry, chemical engineering industry, textile industry, and gold jewelry culture industry of Shanxi Province being mainly concentrated in this study area and these industries being the main pollution sources of these heavy metals [51,53,54].

**Table 3:** The limit values of the heavy metals in the sewage sludge for agricultural use and their mean contents in China (mg·kg^−1^).

	Cu	Zn	As	Hg	Pb	Cd	Cr
USEPA [55]	1500	2800	41	-	300	39	1200
European Union [56] Directive 86/278/EEC	1000–1750	2500–4000	-	16–25	750–1200	20–40	-
Canada [57]	500	2000	10	10	200	20	1000
Netherlands [56]	75	300	15	0.75	100	1.25	75
Austria (Salzburg) [56]	application of sewage sludge and its mixtures is prohibited						
Austria (Tyrol) [56]	application of sewage sludge and products on farmland is prohibited						
Austria (Vienna) [56]	application of sewage sludge is prohibited						
GB 18918-2002 [58]							
pH < 6.5	800	2000	75	5	300	5	600
pH ≥ 6.5	1500	3000	75	15	1000	20	1000
Mean of China [51]	182.5	729.6	11.5	1.4	65.3	2.1	97.5

### 3.2. Assessment of Potential Ecological Risk

According to the Hakanson method, the assessment of the potential ecological risk of sewage sludge for agricultural use is presented in Table 4. By the mean value of $E_{r}^{i}$, heavy metals can be sorted in the following decreasing order: Hg > Cd > Cu > As > Pb > Cr > Zn. This result indicated that the potential ecological risk would be mainly induced by coal-related industries and malleable cast iron production, which were the sources of Hg and Cd [44,48]. Furthermore, for Hg, 7.69% of samples had a high risk, 38.46% had a very high risk, and 53.85% had an extremely high risk; for Cd, 23.08% had a low risk, 61.54% had a moderate risk, and 15.38% had an extremely high risk. These results expressed that all sampled stations had a very low ecological risk from the heavy metals of Cu, Zn, As, Pb, and Cr contained in sewage sludge when the sewage sludge was used on agriculture; however, a large proportion of the stations had a high risk caused by Hg and a moderate risk caused by Cd. Industries such as coal-related industries and malleable cast iron production were the main reasons for the potential ecological risk of the agricultural use of sewage sludge. In addition to these, the jewelry industry might be the other factor for the potential ecological risk by the large production of Hg pollution.

Although the contents of Hg and Cd were the lowest in sewage sludge, because of their highest toxic response factors, they had the greatest responsibility for the potential ecological risk [11,59,60]. Compared to the concentrations of the heavy metals, the heavy metal toxic response factors played an important role in the assessment of monomial potential ecological risk.

To assess the comprehensive potential ecological risks caused by all of the heavy metals in sewage sludge, RI was used. The RI values ranged from 244.77 to 1438.39, and the mean value was 572.51. Based on the value of RI, when sewage sludge was used in agriculture, up to 76.92% of the samples had a high risk or very high risk. From a comprehensive perspective, this result suggests that the agricultural use of sewage sludge was not the suitable disposal method in so many stations in the studied area. To reduce the potential ecological risks caused by the agricultural use of sewage sludge, the contents of heavy metals, especially Hg and Cd, need to be controlled at the source.

**Table 4:** Potential ecological risk assessment results of the use of sewage sludge from heavy metals for agricultural use.

Station	$E_{r}^{i}$							RI
	Cu	Zn	As	Hg	Pb	Cd	Cr	
1	29.00	0.54	11.07	427.12	8.44	33.80	6.01	515.97
2	27.21	0.91	13.55	328.60	6.05	79.37	2.95	458.63
3	16.66	0.74	10.65	425.38	8.17	60.21	4.25	526.06
4	17.80	1.03	11.61	220.80	8.31	55.62	1.76	316.92
5	28.27	0.76	17.31	196.54	6.41	24.94	3.02	277.25
6	14.43	0.57	11.41	583.80	5.50	44.09	2.31	662.11
7	20.06	0.69	12.26	471.30	7.51	71.92	21.46	605.21
8	16.31	0.84	13.02	352.19	6.57	792.52	2.10	1183.55
9	9.85	0.73	9.79	500.59	5.03	27.26	1.33	554.59
10	28.52	0.90	6.92	283.18	6.26	66.13	1.58	393.48
11	18.89	0.71	9.33	171.05	7.61	56.74	1.35	265.68
12	15.37	0.44	6.06	173.84	6.39	41.60	1.07	244.77
13	11.84	7.25	18.00	101.03	3.94	1292.40	3.93	1438.39
Min	9.85	0.44	6.06	101.03	3.94	24.94	1.07	244.77
Max	29.00	7.25	18.00	583.80	8.44	1292.40	21.46	1438.39
Mean	19.55	1.24	11.61	325.80	6.63	203.58	4.09	572.51
SD	12.13	4.74	4.90	230.58	3.18	889.97	1.47	652.25
Low risk	100%	100%	100%	0	100%	23.08%	100%	0
Moderate risk	0	0	0	0	0	61.54%	0	23.08%
High risk	0	0	0	7.69%	0	0	0	46.15%
Very high risk	0	0	0	38.46%	0	0	0	30.77%
Extremely high risk	0	0	0	53.85%	0	15.38%	0	

$E_{r}^{i}$ is the monomial potential ecological risk coefficient of the ith heavy metal; RI is the potential ecological risk index.

### 3.3. Soil Environment Capacity of Heavy Metals

In calculating the soil environment capacity, the risk screening values for the soil contamination for other agricultural land in Soil Environmental Quality–Risk Control Standard for Soil Contamination of Agricultural Land (GB 15618–2018) were used as the heavy metal standard values, except that the corresponding values for As and Hg were chosen from the paddy fields’ standards, which are stricter than those for other agricultural land. The results are shown in Table 5. Based on these results, the heavy metals were ranked in the following decreasing order: Zn > Cr > Pb > Cu > As > Hg > Cd. The soil environment capacities for Zn, Cr, Pb, Cu, As, Hg, and Cd, respectively, were 505.13 kg/km^2^, 423.45 kg/km^2^, 346.95 kg/km^2^, 164.48 kg/km^2^, 22.95 kg/km^2^, 2.19 kg/km^2^, and 1.06 kg/km^2^. The results in study area were basically the same as that those in China [37]. The soil environment capacities of Cu, Zn, Pb, and Cr were relatively higher than those of the others, and Hg and Cr were much lower. This suggested that special attention should be paid to the inputs of Hg and Cd contained in sewage sludge when it is used on agricultural land.

### 3.4. Potential of Agricultural Use of Sewage Sludge

When the heavy metal contents in sewage sludge reached the soil environment capacity, the potential for the agricultural use of sewage sludge was calculated and is presented in Table 6. Due to the different mean potentials of the agricultural use of sewage sludge, heavy metals were ranked in the following decreasing order, Pb > Cr > Zn > As > Cd > Cu > Hg, and the values were 1092.63 a, 808.80 a, 759.9 a, 283.6 a, 203.38 a, 139.14 a, and 131.01 a, respectively. The results suggested that all of the mean potential values far exceeded 10 years, which is the maximum application time specified by the Disposal of Sludge from Municipal Wastewater Treatment Plant–Control Standards for Agricultural Use (CJ/T 309–2009). Pb, Cr, and Zn imposed the loosest restrictions on the agricultural use of sewage sludge. Cu and Hg expressed the toughest restrictions. As and Cd were situated between those two groups. For Zn, Cr, and Pb, this might be because they all had the highest soil environmental capacities. Although Cu had the higher soil environmental capacity, the content of Cu was the greatest in sewage sludge, resulting in the lowest potential. For Hg, the smaller soil environmental capacity gave rise to the smallest potential. This result indicated that Cu and Hg were the limiting factors for sewage sludge’s use in the study area; this was different from that of China as Cd and Zn were the restrictive factors determined by the mean concentrations of the heavy metals [37]. This might have been caused by the large coal industry and coal-related industries in the study area, which would generate large amounts of Cu and Hg pollution [44,48].

The values of potentials ranged from 84.02 to 247.46 a, 78.77 to 1288.44 a, 159.65 to 575.01 a, 57.14 to 330.20 a, 806.02 to 1785.82 a, 8.22 to 425.74 a, and 84.86 to 1696.01 a for Cu, Zn, As, Hg, Pb, Cd, and Cr individually. It can be seen from the above results that only the minimum potential of Cd was less than 10 years. This indicated that the malleable iron industry might be the most crucial limiting factor for the service life period of the agricultural use of sewage sludge in the studied area [63].

The potential of the agricultural use of sewage sludge for different stations ranged from 8.22 to 158.51 a, and the mean value was 81.41 a. This indicated that only one station’s (Station 13) potential for the agricultural use of sewage sludge was below the national maximum application time. Therefore, 46.15% of the stations using sewage sludge for agricultural land were limited by Cu, 38.46% were limited by Hg, and 15.38% were limited by Cd. This situation was not the same as the restrictive factor of Zn, which was calculated based on the mean contents of heavy metals in the sewage sludge of China [37]. This might have been caused by the fact that most industries located in the study area are coal-related industries such as the mining industry, metallurgical industry, and malleable iron industry [39].

**Table 6:** Potential of agricultural use of sewage sludge based on the heavy metal contents in central Shanxi Province (a).

Station	Cu	Zn	As	Hg	Pb	Cd	Cr	N
1	84.02	1061.71	212.68	78.10	806.02	314.22	303.16	78.10
2	89.56	628.49	181.33	101.52	1127.93	133.81	616.68	89.56
3	146.26	774.31	240.26	78.42	837.56	176.38	428.38	78.42
4	136.90	556.14	229.32	151.09	825.35	190.93	1036.37	136.90
5	86.21	752.44	159.65	169.74	1074.03	425.74	602.85	86.21
6	168.90	1007.56	251.16	57.14	1254.21	240.90	788.88	57.14
7	121.46	826.77	242.16	70.78	921.13	147.67	84.86	70.78
8	149.39	678.33	235.97	94.72	1057.09	13.40	865.57	13.40
9	247.46	781.62	324.09	66.64	1382.33	389.63	1367.97	66.64
10	85.45	636.91	473.77	117.80	1114.32	160.59	1152.48	85.45
11	128.98	807.27	362.24	195.03	919.34	187.16	1353.78	128.98
12	158.51	1288.44	575.01	191.90	1099.03	255.31	1696.01	158.51
13	205.72	78.77	199.15	330.20	1785.82	8.22	217.42	8.2
Min	84.02	78.77	159.65	57.14	806.02	8.22	84.86	8.22
Max	247.46	1288.44	575.01	330.20	1785.82	425.74	1696.01	158.51
Mean	139.14	759.90	283.60	131.01	1092.63	203.38	808.80	81.41
SD	86.05	695.04	9.57	178.26	692.83	216.37	60.63	49.41

N is the agricultural use potential of sewage sludge, a.

To sum up, although heavy metals’ contents in sewage sludge were within the standard limits, the agricultural use of sewage sludge in many stations had a high ecological risk, which was induced by the coal-related industries; these industries were also important factors affecting the potential for the agricultural use of sewage sludge. Removing heavy metals from sewage sludge, such as by chemical leaching, bioleaching, electro-kinetic application, and supercritical fluid extraction, in an economic and environmentally and socially acceptable manner is generally restricted by the huge amounts [64,65]. The proper amount of sewage sludge as a fertilizer used on garden plants has no toxic effect on their growth; however, that will have a certain purification effect on the harmful heavy metals in sewage sludge [66,67,68]. This disposal method of sewage sludge is in line with the concept of a circular economy and ecological friendliness; it is expected to become a new way to dispose of sewage sludge.

## 4. Conclusions

Samples were collected from different WWTPs located in central Shanxi Province to assess the ecological risk and potential for the agricultural use of sewage sludge. Contents of heavy metals in sewage sludge were ordered by their mean concentrations as Cu > Zn > Cr > Pb > As > Hg > Cd, and all of them were within the standard limit. The potential ecological risk was assessed, and all heavy metals had a low risk for the agricultural use of sewage sludge, except Hg and Cd, which were the main productions of the coal-related industry that led to a high risk. The values of RI indicated that up to 76.92% of stations had a high or very high risk. The potential ecological risk for the agricultural use of sewage sludge was serious; the Hg and Cd contained in the sewage sludge should be given more attention. The soil environment capacity of heavy metals was evaluated to calculate the potential for the agricultural use of sewage sludge. The mean potentials assessed by the different kinds of heavy metals all exceeded the maximum application time. The potentials of the different stations indicated that only one station’s potential was below the maximum years; Cu, Hg, and Cd, which might have been produced by the coal-related industry, were the limiting factors.

From a comprehensive perspective, coal-related industries, which were the main sources of Cu, Hg, and Cd, crucially affect the agricultural use of sewage sludge. To prevent the heavy metals’ contamination and to make use of nutrients in sewage sludge, using it on garden plants is a good disposal method.

## Figures and Tables

**Table 2:** Correlation coefficients between different heavy metals.

	Cu	Zn	As	Hg	Pb	Cd	Cr
Cu	1						
Zn	−0.333	1					
As	0.037	0.578 *	1				
Hg	−0.100	−0.471	−0.189	1			
Pb	0.402	−0.591 *	−0.316	0.103	1		
Cd	−0.383	0.854 **	0.558 *	−0.378	−0.534	1	
Cr	0.112	−0.021	0.171	0.325	0.251	−0.052	1

Level of significance: * *p* < 0.05, ** *p* < 0.01.

**Table 5:** Soil environment capacities of heavy metal in Shanxi Province.

Statistic	Cu	Zn	As	Hg	Pb	Cd	Cr
Standard value (mg/kg) [61]	100	300	20	1	170	0.6	250
Background value for soil (layer A) of Shanxi (mg/kg) [62]	26.9	75.5	9.8	0.027	15.8	0.128	61.8
Soil environment capacity (kg/km^2^)	164.48	505.13	22.95	2.19	346.95	1.06	423.45
